# Roles of 8-nitro-cGMP in autophagy regulation

**DOI:** 10.1186/2050-6511-16-S1-A14

**Published:** 2015-09-02

**Authors:** Hirokazu Arimoto

**Affiliations:** 1 Graduate School of Life Sciences, Tohoku University, Sendai, Japan

## 

Bacteria-infected cells are known to produce higher level of nitric oxide and reactive oxygen species. Although these reactive chemicals can contribute directly to kill pathogens, in this study, we focus on antibacterial autophagy regulation via downstream mediator. Autophagy is a cellular self-catabolic process in which organelles, biomolecules, and invading pathogens are sequestered in autophagosomes that fuse with lysosomes. However. signaling pathways leading to autophagy activation in innate immunity have not been fully clarified.

**Figure 1:**
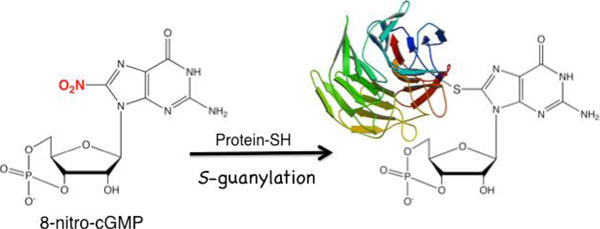
8-Nitro-cGMP modifies proteinous cysteine.

**Figure 2:**
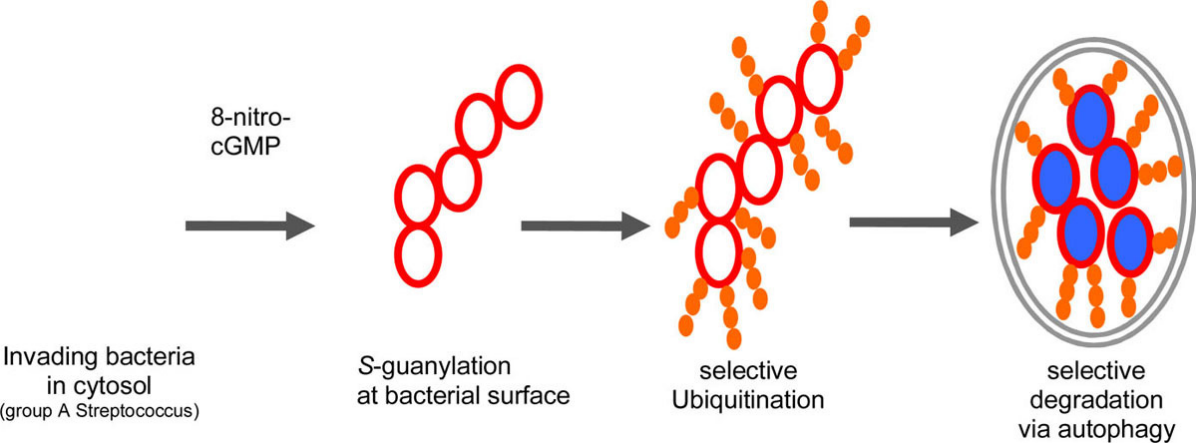
***S*-Guanylation promotes ubiquitination of invading bacteria.** After escape of bacteria from endoosome into the cytosol, their surface is modified by 8-nitro-cGMP via protein S-guanylation, which promotes subsequent Lys63-linked polyubiquitination. Ubiquitin chain is a tag for selective transport of modified cargos to autophagosomes.

A downstream mediator of nitric oxide signaling, 8-nitroguanosine 3’,5’-cyclic monophosphate (8-nitro-cGMP), has been recently identified in various mammalian cell lines [1,2]. This nitrated version of cGMP modifies Cys residues of proteins (protein S-guanylation) to modulate their functions. In this study, we found that endogenous 8-nitro-cGMP promotes autophagic exclusion of invading group A Streptococci (GAS) from murine macrophages [3]. Interestingly, cytosolic GAS were partly modified by the S-guanylation at their surface, and the S-guanylation level was significantly higher with the autophagosome-encapsulated GAS than the cytosolic counterpart. This finding suggests a possible role of S-guanylation as a tag to recruit bacteria for autophagic degradation. Further analysis revealed that S-guanylation-positive bacteria were selectively modified by Lys63-linked polyubiquitin chains, which is a known molecular determinant for selective transport to autophagosomes via autophagy receptor binding. In accordance with this finding, downregulation of S-guanylation suppressed GAS ubiquitination and retarded the clearance of intracellular GAS.

## Conclusion

The results of our study suggested that 8-nitro-cGMP is an endogenous autophagy enhancer that defines targets for sequestration in autophagosomes by recruiting ubiquitin chains.
